# Cochlear size variation among the Argentinian population

**DOI:** 10.1016/j.bjorl.2025.101708

**Published:** 2025-09-10

**Authors:** Maria Fernanda Di Gregorio, Ana Celeste Ferrari, Anandhan Dhanasingh, Maximo Zernotti, Mario Zernotti

**Affiliations:** a Sanatorio Allende, ENT Department, Otology Unit, Cordoba, Argentina; b MED EL Latinoamérica, Buenos Aires, Argentina; c Head of Electrodes Research & Inner Ear Malformations, MED EL Headquarters, Innsbruck, Austria; d Universidad Católica de Cordoba, Cordoba, Argentina; e Universidad Nacional de Cordoba, Cordoba, Argentina

**Keywords:** Cochlear size, Cochlear shape, Cochlear implant electrode

## Abstract

**Objective:**

Cochlear size, shape and the anatomy vary a lot among the human population. Cochlear size is a clinically useful parameter to determine the suitable cochlear implant electrode length. Objective is to assess the cochlear size and shape variation among the Argentine population.

**Methods:**

Computer Tomography (CT) scans of 247 ears were assessed for the cochlear parameters including basal turn diameter (A-value) and width (B-value) and the cochlear height in the mid-modiolar section (H-value) using OTOPLAN® version 4.0. Shape of cochlear basal turn was determined by the ratio between B- and A-values. Cochlear Duct Length (CDL) was estimated from the A-, and B-values.

**Results:**

The A-value was found to vary between 6.7 mm and 10.1 mm with a mean value of 8.7 ± 0.61 mm and the B-value was found to vary between 4.5 mm and 8.1 mm with a mean value of 6.4 ± 0.57 mm. The cochlear height as measured in the axial view from the mid-modiolar section was 3.78 ± 0.57 mm. Shape of the cochlear basal turn as determined by the ratio between B- and A-values showed 41 out of 248 ears (16.5%) have more of a round shaped basal turn with the ratio of ≥0.75 leaving the remaining 207 ears (83.5%) with more of an elliptical shaped basal turn. CDL was found to vary between 24.5 mm and 41.2 mm with a median value of 33.6 mm.

**Conclusion:**

Argentine population appears to have slightly smaller cochlear size compared to population from other countries as reported in literature.

**Level of evidence:**

3.

## Highlights

• Cochlear size is a clinically useful parameter for cochlear implantation.
• Images are crucial to know the exact size of cochlea.
• OTOPLAN® is a software design to 3D reconstruction for inner ear.
• Argentine population appears to have slightly smaller cochlear size.

## Introduction

Clinical medicine has undergone fast, and important paradigm shifts since the beginning of 21st century. Thus, at the end of the 20th century, we moved from medicine based on expert opinion and clinical cases to a revolutionary evidence-based medicine, developed by Paul Shekelle.^1^ The transition from one to the other meant a radical change in the paradigm of approaching pathologies, since evidence became the rule for indicating a medication to address a pathology. This brought more science to daily clinical medical practice, incorporating information from large systematic reviews and meta-analyses.

At this stage, guidelines for the care and approaching of different pathologies began to be written, which until then had been disconcerting due to the variation that existed between the opinions of the experts. The use of guidelines spread improving process and treatments, but the need arose to adapt them to two important factors: the environmental issue (geographical, cultural and economic) and personal variability, which imposed certain limits or changes to them. This trend, without forgetting for a second the evidence-based medicine, forced the development of better and more acute measurement tools to tend towards individualized medicine, a paradigm that must prevail today. In the field of cochlear implants, many important guidelines help us to organize cochlear implants groups up to determine the optimal candidacy.^2^, ^3^, ^4^

Cochlear Implant (CI) is a globally accepted treatment option to restore hearing in sensorineural hearing loss population.^5^ Currently there are three CI manufacturers whose devices are approved by the Food and Drug Administration (FDA) to be commercially available all over the world. The CI has two components, one being the implantable and the other being externally worn audio processor. Sound signal is picked up by the microphone of the audio processor, converted to digital signal by the processor and transferred to the implantable electronics by radio link coil. The implantable electronics converts the digital signal to electrical impulses and is given to the cochlea by the intra-cochlear placement of electrode array.^6^

Electrode array varies in length among the three CI manufacturers to match with the cochlear size variation. There are almost 17 different electrode arrays in straight configuration and 3 in pre-shaped configuration commercially available from all 3 FDA approved CI manufacturers.^7^ Straight electrodes vary in length from a minimum of 15 mm to a maximum of 34 mm. Hearing outcomes in patients with CI is multifactorial as reported in several studies and electrode angular insertion depth as one of the factors appears to have some effect on the clarity of hearing and overall hearing scores when tested in noisy environment in post-lingual deaf patients.^8^ Reason for this finding is closely associated with a good match between tonotopic frequency and electrode position inside the cochlea with deep insertion, wider contact separation between electrode channels and application of fine structure processing of audio signal to the low frequency regions inside the cochlea.

This means for optimal outcomes; CIs must be tailored to each recipient's requirements. Earlier studies have shown that cochlear size variation can be up to 40% deviating from the average size.^9^ Cochlear size is widely known to be measured by the A-value, which is the diameter of cochlear basal turn in the oblique coronal view or the cochlear view.^10^,^11^

Khurayzi et al. did a literature review on the cochlear size variation as measured by A-value and found 26 articles reporting on average ranging from a minimum of 7.9 mm to a maximum of 10.2 mm.^12^ The A-value is directly proportional to the full length of cochlea as measured along the organ of Corti as reported by Alexiades et al.^9^ or along the outer wall as reported by Escude et al.^13^

Clinical applications of Cochlear Duct Length (CDL) include choosing appropriate electrode length, creating cochlear size based frequency map following Greenwood’s equation, and identifying the angular depth at which the low frequency residual hearing if present. Dhanasingh et al. demonstrated the applications of CDL through a research software which was later developed as OTOPLAN® which is CE marked and FDA approved to be used clinically.^14^ The software further shows the breakdown of CDL into basal turn length, second turn length and the apical turn length separately making us to be conscious about the importance of stimulating the second turn of cochlea which is crucial for better results in the perception of the speech. Knowing that average CDL is approximately 35 mm, inserting electrodes of lengths 20, 23, and 24 mm would only cover the basal turn of cochlea resulting in a gross error in cochlear tonotopy.

Although there are already numerous studies that determine values of cochlear ducts longer than the electrodes we usually use, still many surgeons insist the cochleae are on average smaller in their populations, based only on personal experiences or only to justify the election of short electrodes. For this reason, we wanted to develop the first study of cochlear size measurement in the Argentine population, as a first step to extend this to South American population (In general with the same genetic origin). It should be noted that the Argentine population constitutes a typical example of a highly variable ethnic origin, since according to different authors, the ethnical composition of Argentina’s population is heterogeneous.^15^ Avena SA et al., reported in 2001 that the gene mixture average population of Argentina contains 79.9% (±0.4) of European contribution, 15.8% (±0.4) Indigenous and 4.3% (±0.2) African, while Seldin et al. confirmed that the average genetic structure of Argentinian population contains 78% of European contribution, a 19.4% indigenous and 2.5% African.^16^,^17^

Clinical data from South American population is still not widely reported in literature and therefore the aim of this study is to understand the overall cochlear size variation from the Argentine population. Measuring the cochlear size reliably without much of human error is essential and therefore we applied OTOPLAN®, a CE marked medical image analysis software specially designed for inner ear application.

## Methods

**Fig. 1:**
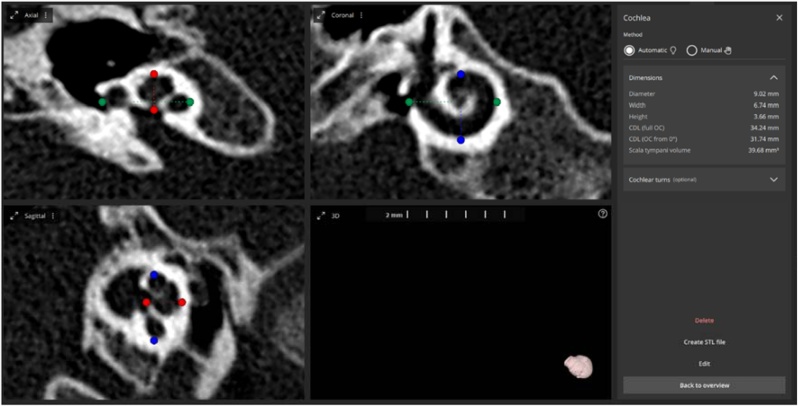
Screenshot of OTOPLAN® version 4.0 showing the cochlea in three different planes and the cochlear parameters.

Pre-operative Computer Tomography (CT) images of CI candidates from our center between 2022 and 2024 were retrospectively taken for cochlear size assessment. Attention was given to exclude those cases that had abnormal inner ear conditions including inner ear malformations, ossification due to meningitis and otosclerosis to avoid error in the A-, B-, and H-value measurements. OTOPLAN® version 4.0 was used in this study to automatically measure the cochlear size by simply uploading the CT scans and clicking the “Create 3D model” button as shown in Fig. 1. Slice thickness of CT scans taken for this study was found to be ≤ 0.6 mm which was just good for automatic measurement of cochlear size by the software. If these parameters were to be measured manually, then the A-value or in other words the largest diameter of cochlear basal turn is measured by a straight line connecting the RW entrance and the opposite side of lateral wall passing through the center point of cochlea in the cochlear view/oblique coronal plane showing the cochlear basal turn as shown by green dotted line in Fig. 1. The B-value or in other words the width of cochlear basal turn is the line drawn perpendicular to the A-value line in the cochlear view as shown by blue dotted line in Fig. 1. The H-value or cochlear height is captured in the axial view cutting through the cochlear mid-modiolar section measuring from the base to apex as shown by the red dotted line in Fig. 1. OTOPLAN® version 4.0 gives the possibility to make these measurements automatically without introducing human error from manual measurement. Our center started using OTOPLAN® version 4.0 in clinical practice in 2022 and this was the reason to consider CT scans only from 2022 till 2024 for this retrospective study.

Shape of the cochlear basal turn is assessed by the ratio between the B- and A-value. A higher ratio is considered as round shaped basal turn whereas a lower ratio corresponds to an elliptical shaped basal turn as per Khurayzi et al.^12^

## Results

**Fig. 2:**
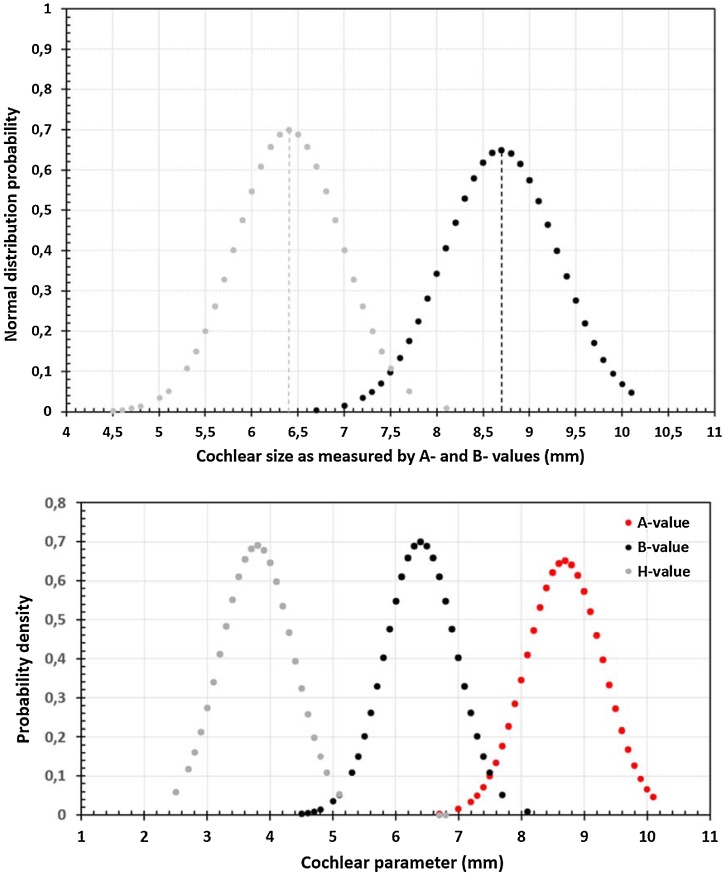
Normal distribution probability of A-, B-, and H-values of cochleae from Argentine population.

A total of 248 CT scans with normal anatomy inner ear underwent OTOPLAN® version 4.0 analysis for the automatic assessment of cochlear size. A total of 20 CT scans showed some degree of inner ear malformations, otosclerosis, and ossification and were excluded from the study. The A-value was found to vary between 6.7 mm and 10.1 mm with a mean value of 8.7 ± 0.61 mm and the B-value was found to vary between 4.5 mm and 8.1 mm with a mean value of 6.4 ± 0.57 mm. The cochlear height as measured in the axial view from the mid-modiolar section was 3.78 ± 0.57 mm. Fig. 2 shows the normal distribution probability of A-, B-, and H-values. A total of 183 ears out of 248 ears (74%) were found to have either average or above average cochlear size as measured by the A-value. Twelve ears were measured with the average cochlear size value of 8.7 mm also making it the median value.

**Fig. 3:**
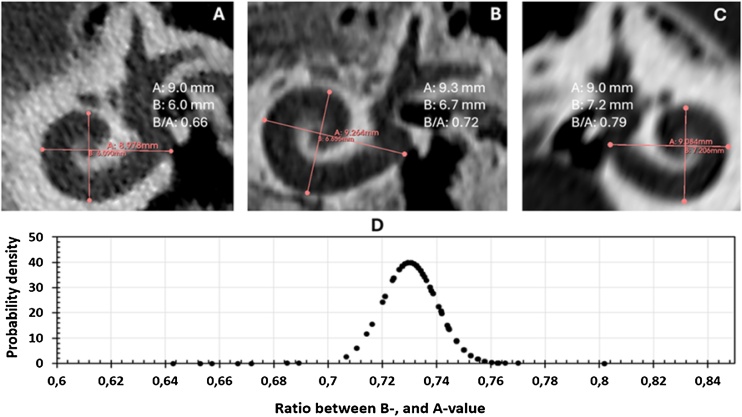
Shape of cochlear basal turn. Elliptical shaped basal turn (A), close to a round shaped based turn (B), round shaped basal turb (C) and probability density of the shape of cochlear basal turn (D).

Shape of the cochlear basal turn as determined by the ratio between B- and A-values showed 41 out of 248 ears (16.5%) have more of a round shaped basal turn with the ratio of ≥ 0.75 leaving the remaining 207 ears (83.5%) with more of an elliptical shaped basal turn. Fig. 3A and 3B shows two cochlear samples that are more of elliptical shaped basal turn whereas Fig. 3C with being more of a round shaped basal turn. The ratio ranged between 0.64 and 0.802 as shown in Fig. 3C and with a median value of 0.73.

**Fig. 4:**
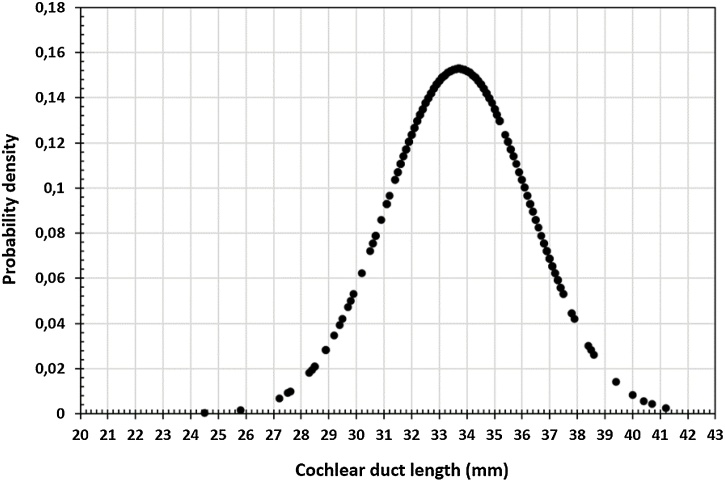
Distribution of Cochlear Duct Length (CDL) as estimated from A-, and B-values.

Distribution of CDL as estimated from A- and B-value is shown in Fig. 4. The CDL from Argentine population ranges from 24.5 mm and 41.2 mm with a median value of 33.6 mm. Table 1 shows the breakdown of CDL values and number of ears identified with CDL under 30 mm, between 30 and 35 mm, and above 35 mm.

**Table 1:** Number of ears measured with certain CDL.

CDL (mm)	Number of ears
≤30	19
>30 and ≤35	157
>35	72

## Discussion

Cochlear size variation among the human population is a widely studied topic in the CI field since 1938.^18^ A simple PubMed search on the term “cochlear size” fetches a total of 2386 articles at the time of writing this paper shows how well this topic is familiar in the CI field. To the best of our limited knowledge after a thorough literature search not a single report on the cochlear size from Argentine population was found and that motivated us to perform this study.

Compared to literature reports on cochlear size as measured by A-value ranging between 7.9 mm and 10.2 mm with an average value of 9.1 mm, our population cochlear size appears to be on the smaller size ranging between 6.7 mm and 10.1 mm with an average value of 8.4 mm. OTOPLAN® also provides the CDL estimation based on the A- and B-values which could be useful for electrode length selection. All these information about the cochlea is clinically useful when comes to patient counselling and electrode length selection. Shape of the cochlear basal turn is another interesting parameter that needs to be studied in relation to the ease of electrode insertion. We assume that a relatively round shaped based turn should offer an easy insertion of straight electrode than an elliptical shape basal turn due to electrode making a tight turn at around 180° of angular insertion depth in the latter case.

There are many studies on CDL from different regions of the world, representing different ethnic origin. Chen et al. reported an average CDL of 34.37 ± 1.481 mm in Chinese population, while Usami et al.^19^ from Japan reported an average CDL of 35.1 ± 1.6 mm in Japanese population.^20^ An Arabian report showed statistical significance differences in CDL measured between males and females, with an average CDL of 33.39 ± 1.52 mm in males and 32.47 ± 1.90 mm in females.^21^ An European study published mean CDL of 36.2 ± 1.8 mm with significant differences between genders (female: 35.8 ± 0.3 mm; male: 36.5 ± 0.2 mm; *p* = 0.037), but none concerning side or age.^22^ Compared to all these literature findings, we report from the Argentine population that the average and median CDL is 33.6 mm which is smaller than Chinese, Japanese and European reports.

These data show that in different ethnics, the CDL is longer than 31 mm. To achieve better speech performance with CI, measuring the patient's CDL and choosing an appropriate CI electrode length seems to be important. Many reports showed the presence of Spiral Ganglion Cells Bodies (SGCBs) beyond the second turn. Dhanasingh et al. in a literature review shows SGCBs inside the Rosenthal's canal in the modiolar trunk extended to an angular depth of 630°‒680°, which is close to the end of the second turn of the cochlea.^23^ Agrawal et al. in 2020 published a cornerstone paper on this topic with Synchrotron high radiation CT images determining the presence of SGCBs up to the second turn of cochlea.^24^ This point is crucial in determining how far the electrical stimulation should be provided inside the entire cochlea. A strong positive correlation exists between the SGCB numbers in the 4th (apical) segment with better speech-discrimination scores in individuals with normal hearing, as per Otte et al.^8^^,^^25^ Bringing all these evidence on the presence of SGCBs up to 650° of angular depth and the CDL distribution from Argentine population in to clinical perspective, we recommend a 24 or 26 mm long electrodes as a suitable electrode choice for CDLs that are ≤ 30 mm followed by a 26, 28 and 31 mm long electrode for CDLs between 30 and 35 mm. For those CDLs that > 35 mm, a 31 or 34 mm long electrode would be an ideal choice.

All reports in literature on cochlear size measurement up until 2023 involved manual measurement of cochlear size using different DICOM viewers including previous versions of OTOPLAN®, and the amount of human error cannot be ignored. Since 2024, the version-4 of OTOPLAN® offers the possibility to automatically measure the cochlear size that assisted us to perform this study in a relatively quick time with zero degree of human error. It takes a minute to upload a CT scan and automatically measure the cochlear size in OTOPLAN® which needs to be highlighted here.

One of the key limitations of this study was the selection of CT scans only from the last two years between 2022 and 2024 which could affect the cochlear size range and average size as reported from the Argentine population in this study. Our future work is to prospectively apply OTOPLAN® in both cochlear size assessment and electrode length selection and to evaluate the effect of basal turn shape on the ease of electrode insertion.

## Conclusion

In conclusion, based on the findings of this study, the Argentine population appears to have slightly smaller cochlear size compared to population from other countries as reported in literature. OTOPLAN version 4.0 is a clinically useful tool in automatically measure the cochlear size without involving human error. Therefore, the preoperative measurement using OTOPLAN® is highly recommended in selecting the appropriate electrode array length based on the individual's CDL.

## ORCID IDs

Maria Fernanda Di Gregorio: 0000-0001-9963-8343

Ana Celeste Ferrari: 0009-0008-1943-5350

Anandhan Dhanasingh: 0000-0003-2116-9318

Maximo Zernotti: 0000-0003-3616-0324

Mario Zernotti: 0000-0003-4288-2809

## Funding

There is not funding resources for this investigation.

## Declaration of competing interest

Maria Fernanda Di Gregorio, Maximo Zernotti and Mario Zernotti declare have not conflict of interest.

Ana Celeste Ferrari and Anandhan Dhanasingh are full time employees of MED-EL GmbH.
